# Loss‐Engineered Terahertz Pixelated Metasensors for Enhanced Molecular Vibrational Fingerprint Sensing

**DOI:** 10.1002/advs.78233

**Published:** 2026-10-14

**Authors:** Chenglin Yang, Yuqing Cui, Donghai Han, Yushan Hou, Zhonglei Shen, Andreas Tittl, Liuyang Zhang, Ruqiang Yan, Xuefeng Chen

**Affiliations:** ^1^ School of Mechanical Engineering Xi'an Jiaotong University Xi'an Shaanxi China; ^2^ Division of Physics and Applied Physics Nanyang Technological University Singapore Singapore; ^3^ Institute of Photonics Hamburg University of Technology Hamburg Germany; ^4^ Chair in Hybrid Nanosystems Nanoinstitute Munich Faculty of Physics Ludwig‐Maximilians‐Universität München Munich Germany

**Keywords:** 3D printing, light‐matter interactions, molecular vibrational sensing, pixelated metasensor, terahertz spectroscopy

## Abstract

Molecular vibrational fingerprints in the terahertz (THz) regime offer unique opportunities for label‐free chemical and biological sensing. Metasensors can amplify these vibrational signals through local near‐field enhancement. However, current THz molecular fingerprint metasensors lack a systematic design and optimization methodology, which severely limits their potential. Here, we unveil the crucial role of resonant loss rate in THz molecular fingerprint sensing and present a loss‐engineering methodology for optimized molecular vibrational sensing. Based on the proposed methodology, we experimentally demonstrate a 12.6‐fold enhancement of the molecular fingerprint signal with three‐dimensionally printed pixelated metasensors.  Furthermore, the pixelated metasensors support vibrational strong‐coupling, providing a unified platform for enhanced molecular sensing and strong light‐matter interaction. Overall, the proposed metasensor could bridge THz molecular vibrational fingerprint sensing to end‐use applications in label‐free chemical and biomedical detection.

## Graphical abstract

This work presents a THz pixelated metasensor that employs loss optimization and spectral overlap for enhanced molecular vibrational fingerprint sensing. The flexible loss rate tuning through the out‐of‐plane dimension allows the device to operate in the optimal fingerprint enhancement condition. The multi‐pixel design further broadens spectral coverage, demonstrating a scalable route toward high‐performance THz molecular sensing.

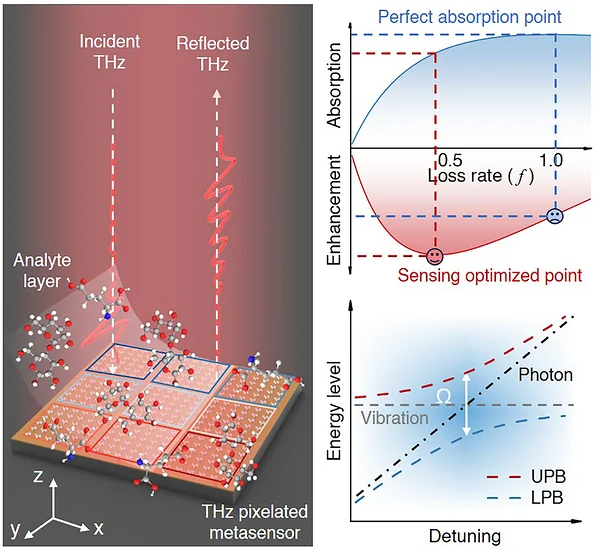

## 1 Introduction

Terahertz (THz) spectroscopy has emerged as a powerful technique for molecule‐specific analysis because it directly probes the low‐energy vibrational and rotational modes of molecules [1, 2, 3]. Compared to other vibrational spectroscopy techniques, such as Fourier‐transform infrared (FTIR) [4, 5, 6, 7] and Raman spectroscopy [8, 9, 10], THz spectroscopy offers deeper penetration, non‐destructive detection, and high sensitivity to intermolecular interactions and collective molecular dynamics. Thus, it has become an ideal tool for identifying gases [11], liquids [12, 13, 14], biomolecules [15, 16, 17, 18], and cancer cells [19, 20, 21]. However, the practical sensing performance of THz fingerprint spectroscopy is hindered by inherently weak THz‐matter interactions, which stem from the large mismatch between sub‐millimeter‐scale THz waves and nanoscale molecular vibrational modes [22, 23]. To overcome these limitations, artificially structured metasurfaces that confine and amplify electromagnetic fields in subwavelength volumes [24, 25, 26] have been extensively employed to enhance THz‐matter interactions [27, 28, 29].

The sensing performance of metasurface‐based molecular fingerprint systems is primarily governed by two key factors: spectral overlap and light‐matter coupling strength [30, 31, 32]. Recent research on THz molecular vibrational fingerprint enhancement has mainly focused on designing multi‐resonant or broadband metasurfaces to match the rich vibrational fingerprint characteristics of molecules in the THz region [33, 34, 35, 36], as the enhancement reaches its maximum when the resonance and the vibrational frequencies are spectrally aligned [37]. For instance, by introducing multi‐resonant unit cells [38, 39], frequency‐tunable designs [40, 41], or pixelated arrays [33, 42], metasurfaces can be engineered to support multiple resonances within a single device [30, 43]. Although these approaches address spectral overlap, further performance gains require optimized light‐matter coupling strength.

Despite the progress in spectral matching, enhancing the coupling strength in the THz regime remains challenging, even though it is crucial for trace‐substance detection and for reaching the vibrational strong‐coupling regime. Current strategies typically rely on molecular enrichment [44, 45], nanoscale hot‐spot construction [22, 46], and near‐field spectroscopy detection [47]. Molecular enrichment aims to maximize the overlap between analyte molecules and the electromagnetic hot spots through surface functionalization or physical trapping, nanoscale hot spots enhance local electric fields by reducing structural dimensions to tens or hundreds of nanometers, and near‐field detection compensates for the exponential decay of evanescent signals. However, these approaches often increase fabrication complexity, reduce device robustness, or impose demanding detection conditions, and none of them fundamentally solves the problem of weak THz wave‐matter interaction. An alternative and more intrinsic route for improving light‐matter coupling strength lies in engineering the loss rate of resonant systems, defined by the ratio of radiative to intrinsic losses [48].

In principle, the loss rate of a metasurface resonator can be tailored by tuning structural parameters. However, conventional planar lithography confines this tuning to in‐plane parameters, such as the period or resonator length, which mainly shift the resonance frequency while having little influence on the balance between radiative and intrinsic losses. This limitation arises because such parameters modify the surface geometry rather than the internal field distribution. Recent advances in three‐dimensional printing (3D‐printing) now allow metasurfaces with out‐of‐plane geometries to be fabricated with precise control over the resonator height [49, 50, 51, 52]. However, such 3D‐printed architectures have not yet been explored systematically for loss‐rate tuning or for enhancing THz molecular fingerprint sensing.

In this work, we present loss‐engineered THz pixelated metasensors that simultaneously achieve spectral overlap and coupling optimization for enhanced molecular vibrational sensing. Notably, by introducing an out‐of‐plane degree of freedom, the resonance frequency and loss rate of pixelated metasensors can be controlled independently. This new out‐of‐plane degree of freedom alters the cavity volume and field confinement, enabling concurrent manipulation of radiative and absorptive losses and allowing the metasensor to operate under optimized coupling conditions for maximum molecular vibrational enhancement. The out‐of‐plane metasensors are fabricated in a three‐step process based on projection micro‐stereolithography (PµSL). Most importantly, we experimentally demonstrate that the metasensor delivers a 12.6‐fold improvement in sensing sensitivity when reconstructing the molecular fingerprint of maltose. Finally, we achieve vibrational strong coupling between the metasensor and glutamic acid, and reveal how the coupling strength evolves from the weak to the strong regime. Together, these results highlight the importance of loss engineering in THz sensing and establish a route to high‐sensitivity, broadband biochemical sensing.

## 2 Results and Discussion

### 2.1 Working Mechanism of the THz Pixelated Metasensor

The concept of the THz pixelated metasensor is illustrated in Figure 1a. Blood‐based biomarker detection is central to biomedical testing, since it underpins the diagnosis of a wide range of diseases. Conventional analytical techniques, such as enzyme‐linked immunosorbent assays (ELISA) and liquid chromatography mass spectrometry (LC‐MS), often involve multistep sample preparation, long processing times, and bulky instrumentation, as shown in the upper right panel of Figure 1a. In contrast, THz metasensor‐based detection exploits the characteristic vibrational fingerprints of biomarker molecules in the THz regime. These molecular signatures are strongly amplified by the metasensor, enabling highly sensitive readout. Moreover, the pixelated design of the metasensor provides broadband enhancement, allowing simultaneous detection of different biomarkers within a single device. This approach thus offers a fast, convenient, and potentially high‐throughput alternative for biomolecular screening, as shown in the lower right panel of Figure 1a.

**FIGURE 1:**
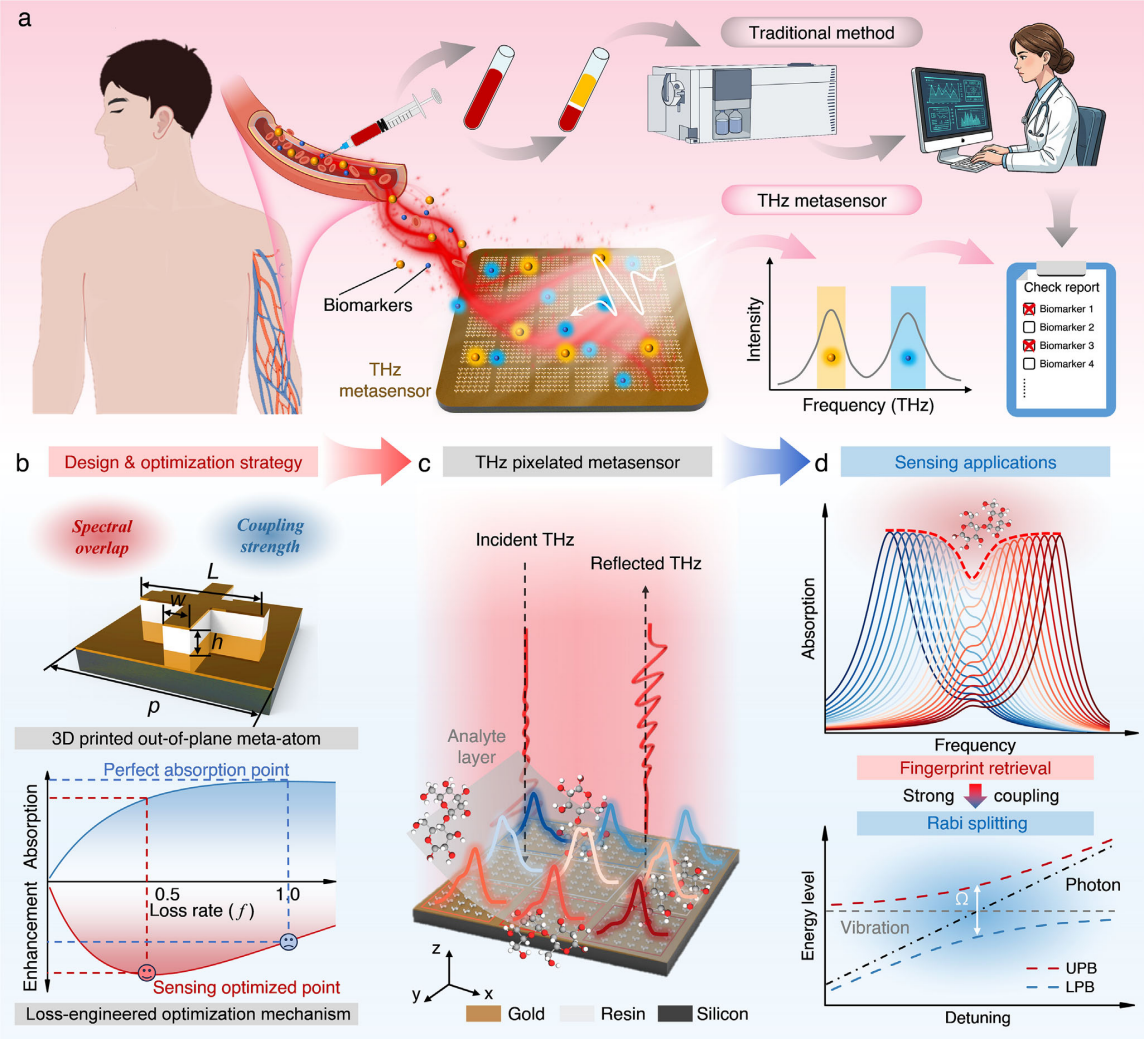
Working mechanism of the loss‐engineering optimized THz pixelated metasensor. (a) Schematic illustration of the traditional method and THz metasensor‐based method for biomarker detection. (b)‐(d) Operating principle of the THz metasensor. (b) The upper panel shows a schematic of the meta‐atom, which can be fabricated by 3D printing. The lower panel shows the loss‐engineered sensing performance optimization mechanism, in which the optimized sensing point can be achieved by tailoring the loss rate *f* of the resonator, where *f* is defined as *f* = *γ*_r_/*γ*_a_. (c) Schematic of the metasensor consisting of a 3×3 array of reflective meta‐pixels. With biomarkers covering the metasensor, the reflected THz signals of the pixels encode the characteristic molecular vibrational information. (d) Sensing applications of the proposed metasensor, from molecular fingerprint retrieval to vibrational strong coupling.

The operating principle of the metasensor is shown in Figure 1b–d. As shown in the upper panel of Figure 1b, the meta‐atom is designed as a cross‐shaped resonator with an out‐of‐plane configuration [33, 53, 54], in which gold top and bottom layers are separated by a dielectric spacer. Notably, the bottom metal layer is thicker than the THz skin depth, which suppresses transmission and makes the device operate in reflection. When the linearly polarized THz wave is incident, the electric dipole resonance can be excited, resulting in electrical hotspots at both ends of the meta‐atom, and the maximum electric field enhancement can reach the order of 10^4^ (Figure S1). When molecular layers are loaded onto the metasensor, the pixels can function as THz probes to amplify molecular signals, and the enhanced vibrational spectrum can be observed via far‐field responses. This process is generally called the plasmon‐phonon coupling effect, and the coupling strength is directly proportional to the overlap of the resonance and the vibrational fingerprint [55]. When the resonant frequency of the pixel matches the molecular vibrational frequency, molecular absorption produces the largest modulation of the far‐field response of the pixel. This is known as the spectral overlap effect [39]. In our design, by tuning the resonator length *L* of the meta‐atom, the far‐field responses of metasensors can cover a wide frequency range. Therefore, metasensors with a multi‐pixel configuration overcome the problem of spectral detuning.

In the sensing system, the THz‐matter coupling strength is a key parameter that influences the magnitude of enhancement for molecular vibrational signals [56, 57]. For a given resonator‐molecule system, one particular loss rate (*f*), set by the out‐of‐plane dielectric layer, provides the maximum coupling efficiency and the largest enhancement of the molecular vibration signal, outperforming the perfect‐absorption point, as shown in the lower panel of Figure 1b. Benefiting from its multi‐pixel design and the ability to tailor the coupling strength, the metasensor is particularly well suited to enhancing molecular vibrational fingerprints and to realizing vibrational strong coupling, as shown in Figure 1d.

### 2.2 Loss Tuning Strategy of the Cross‐Shaped Meta‐Atom

To optimize the sensitivity of the metasensor by loss engineering, we first analyze how loss affects the resonance. According to TCMT [58, 59], the metasurface can be treated as a one‐port single‐mode system because of the ground metal layer (Figure 2a). The resonance absorption of the metasurface *A*_max_ can be defined as (see Section S2 for the detailed derivation):

(1) $$A_{\max}=\frac{4\gamma_{r}\gamma_{a}}{{\left(\gamma_{r}+\gamma_{a}\right)}^{2}}=\frac{4f}{{\left(1+f\right)}^{2}}$$

where *γ*_r_ and *γ*_a_ represent the radiative and intrinsic losses, respectively. The loss rate *f* is defined as *f* = *γ*_r_/*γ*_a_. According to Equation (1), the resonance absorption of the metasurface can be determined by the loss rate *f* (Figure 2b). Perfect absorption occurs under the critically coupled (CC) condition when *f* = 1, yielding a maximum absorption of unity. For *f* ≠ 1, the imbalance between radiative and intrinsic losses drives the system into either the over‐coupled (OC) or under‐coupled (UC) regime, depending on which loss dominates [23, 53, 60, 61].

**FIGURE 2:**
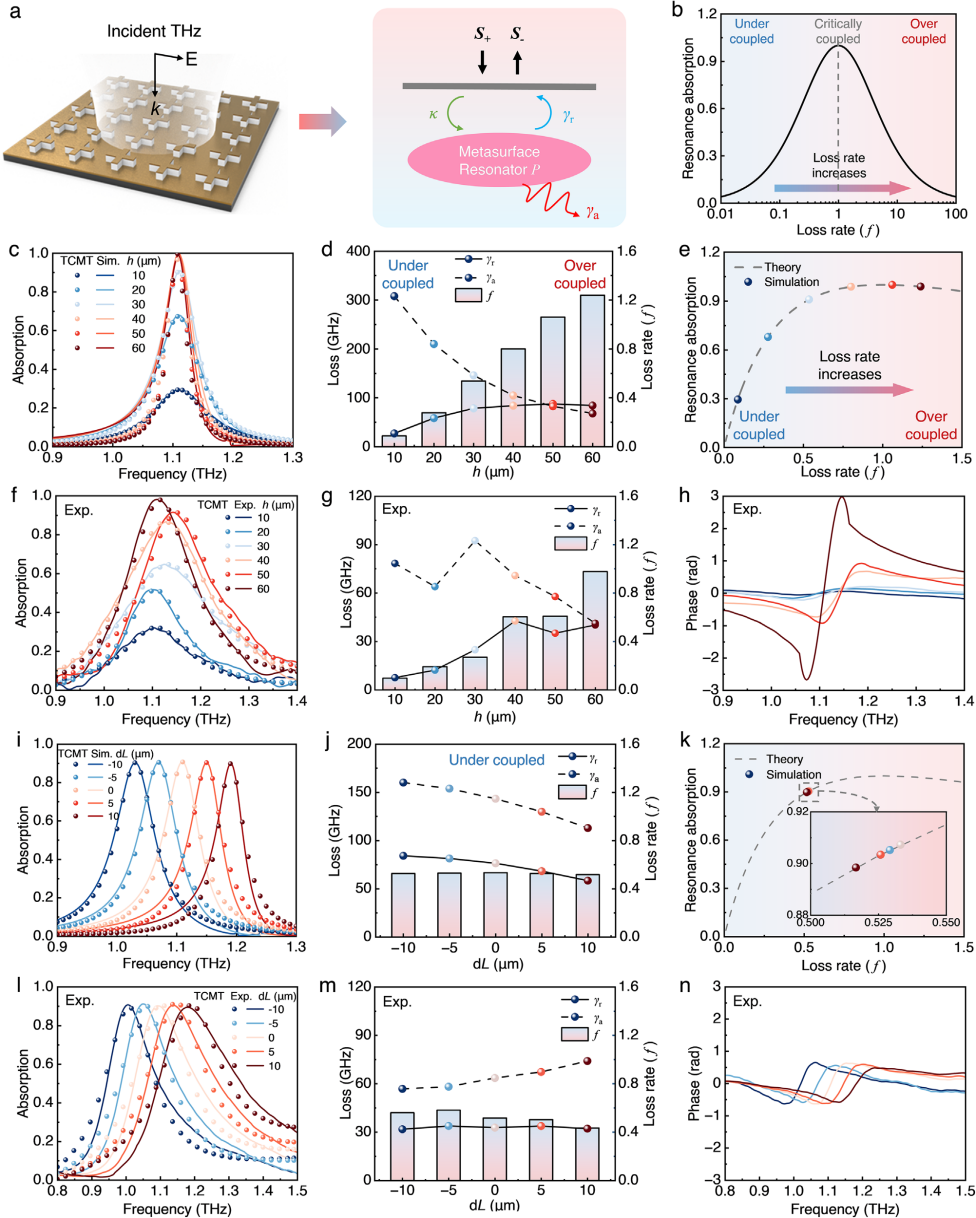
Loss tuning strategy of meta‐atoms. (a) Illustration of the metasurface and its TCMT model. The meta‐atom can be modeled as a one‐port single‐mode system owing to the presence of back reflector. (b) Resonant absorption of the meta‐pixel as a function of loss rate *f*. Both the absorption and coupling states can be readily controlled by *f*. Simulated (c) absorption spectra and TCMT fits, (d) extracted loss rates, and (e) resonant absorption for different *h* values. Experimental (f) absorption spectra and TCMT fits, (g) extracted loss rates, and (h) phase spectra for different *h* values. Simulated (i) absorption spectra and TCMT fits, (j) extracted loss rates, and (k) resonant absorption for different d*L* values. Experimental (l) absorption spectra and TCMT fits, (m) extracted loss rates, and (n) phase spectra for different d*L* values.

After understanding the key role of the loss rate *f* in determining the response of metasurfaces, we now focus on linking the structural parameters of the meta‐atoms to it. Figure S3a shows the absorption spectra for different *h*, along with their TCMT fitting results. The resonance absorption increases substantially with increasing h, accompanied by a gradual redshift of the resonance frequency. Using TCMT, the resonance parameters are extracted as shown in Figure S3d. This reveals that the increase of the absorption is primarily due to the distinct dependence of radiative and intrinsic losses on the out‐of‐plane dimension: the radiative loss *γ*_r_ increases with increasing *h*, while the intrinsic loss *γ*_a_ decreases rapidly. Two physical mechanisms underpin this behavior. On the one hand, *γ*_r_ is related to the coupling efficiency between the resonator and free space. A thicker resonator modifies the near‐field distribution and enhances its overlap with free‐space radiation channels, which enlarges the scattering cross‐section and the dipole moment and causes a modest increase in radiative loss *γ*_r_. On the other hand, *γ*_a_ is related to the energy storage within the resonator. To further explain this, we define the intrinsic absorptive quality factor *Q*_a_ as the ratio of the total energy *U* stored in the resonator cavity during one oscillation period to the energy absorbed by the medium spacing layer *P*_d_ and the metal *P*_m_, that is: *Q*_a_ = *ω*_0_ *U*/(*P*_d_+*P*_m_) [62], where *ω*_0_ is the resonance frequency. As the height of the dielectric layer of the resonator increases, the total stored energy *U* increases, and this increasing trend is greater than the rate of the increase of losses in the dielectric *P*_d_ and in the metal *P*_m_. In other words, the relative weight of energy dissipation decreases, which will lead to an increase in *Q*_a_. According to the relationship between *Q*_a_ and *γ*_a_: *Q*_a_ = *ω*_0_/*γ*_a_, this will lead to a decrease in *γ*_a_. Consequently, the ratio between *γ*_r_ and *γ*_a_ can be effectively tuned by varying the resonator height *h*, resulting in a wide range of accessible loss rates. The resonator height is therefore an efficient parameter for engineering the coupling regime of the metasurface.

Next, we investigate how the in‐plane structural parameters, the resonator length *L* and the period *p*, affect the loss rate *f*. An increasing *L* leads to a continuous redshift of the resonance frequency, with the frequency varying almost linearly with *L* (Figure S3b). Although the resonance absorption also changes slightly during this process, the extracted loss rates indicate that the influence of *L* on the loss is relatively weak compared to that of *h* (Figure S3e). Figure S3c,f shows the effect of varying the period *p* on the absorption and the loss. The modulation of the loss rate *f* caused by the change of *L* and *p* could be related to changes in the resonator filling fraction. Increasing *L* or decreasing *p* increases the proportion of the resonant structure within each unit cell, leading to a denser resonator array and stronger electromagnetic interaction with the lossy structural materials. As a result, both intrinsic and radiative losses are enhanced, with the radiative loss showing a stronger relative variation, thereby increasing the loss rate *f*. Overall, compared to the dielectric layer thickness *h*, the in‐plane structural parameters *L* and *p* have a much smaller impact on the loss rate because their influence on the radiative and intrinsic losses is constrained by the geometry of the surface, rather than the internal cavity structure. Consequently, the available tuning range through in‐plane parameter adjustments is limited.

The above analysis yields a strategy for tuning the loss rate and the resonance frequency continuously and independently through the three parameters *h*, *L*, and *p*. First, the variation of *h* predominantly sets the loss rate of the resonance. Second, by tuning *L*, a multi‐resonance pixelated metasensor can be constructed, and the problem of spectral detuning is thereby overcome. Finally, fine‐tuning the period *p* helps to address the minor changes in loss rate introduced during the tuning of *L*. Based on the above strategy, two special cases can be realized: one is a series of resonators with the same resonant frequency but different loss rates, and the other is a resonant series with stable loss rates but changing resonant frequencies. The former is valuable for studying molecule‐resonator coupling systems, as it allows the investigation of coupling systems under conditions of spectral matching to optimize the resonators. The latter is advantageous for molecular fingerprint reconstruction and vibrational strong coupling, since it allows the spectral overlap to be tuned.

Figure 2c shows the absorption spectra with different loss rates, while their resonant frequencies are tuned to around 1.11 THz by numerically optimizing the parameters *L* and *p*. As the height *h* increases from 10 to 60 µm, the radiative loss *γ*_r_ shows a slight increasing trend, while the intrinsic loss *γ*_a_ decreases sharply (Figure 2d), which leads to an increase in the loss rate *f*, transitioning from an under‐coupled mode to an over‐coupled mode, and the resonant absorption increases with the increasing loss rate (Figure 2e), in agreement with the theoretical results calculated by Equation (1). To experimentally validate the mechanism, we fabricated the samples and measured their absorption spectra. The corresponding experimental results are presented in Figure 2f. Owing to fabrication tolerances associated with the 3D‐printing process, the measured resonance frequencies exhibit a slight deviation from the designed value of 1.11 THz. Nevertheless, the overall spectral evolution agrees well with the theoretical prediction. As the resonator height increases, the absorption amplitude rises markedly, in accordance with the simulated results. To quantitatively analyze the coupling condition, the measured absorption spectra were fitted using TCMT (Section S2). As shown in Figure 2f, excellent agreement is obtained between the experimental spectra and the TCMT fitting results, confirming the validity of the proposed theoretical framework. Based on the fitted parameters, the radiative loss *γ*_r_ and intrinsic loss *γ*_a_ were extracted for each device. The experimentally obtained loss evolution shows the same trend as the numerical simulations. Furthermore, to independently verify the coupling state of the fabricated devices, we reconstructed the reflection phase spectra from the experimentally measured complex reflection fields, as shown in Figure 2h. The phase excursion of the devices progressively increases and exhibits a pronounced near‐2π transition near critical coupling for *h* = 60 µm (Figure 2h).

To obtain a series of resonances with a stable loss ratio and different resonant frequencies, *L* and *p* need to be optimized simultaneously. The tuning of the resonance frequency and the loss rate *f* by *L* and *p* is intrinsically coupled, since both quantities vary with these two geometric parameters. However, collaborative adjustment of *L* and *p* enables an approximate decoupling between the resonance frequency and loss ratio, yielding a series of resonances with nearly constant *f* while the resonance frequency varies approximately linearly. Importantly, variations in *L* and *p* induce opposite changes in the loss ratio *f* (Figure S3). Therefore, a balancing factor *S* is introduced to compensate for their respective effects on the loss ratio. Specifically, *p* and *L* can be determined by:

(2) $$\begin{array}{ccc} L & = & L_{0}-dL\\ p & = & p_{0}-S*dL \end{array}$$

where *L*_0_ and *p*_0_ are the reference length and period. Physically, *S* represents the relative compensation between the influences of *L* and *p* on the resonator filling fraction and the associated radiative–intrinsic loss balance. By appropriately combining *L* and *p*, their loss‐tuning effects can partially cancel while the resonance frequency is continuously adjusted. For any given *h*, a stable loss rate is obtained by adjusting the balancing factor *S*. Figure 2i shows the absorption spectra with the same height *h* = 30 µm, while their resonant frequencies are tuned to cover a wide bandwidth range. Figure 2j shows the extracted loss of the resonances. As d*L* varies from −10 to 10 µm, both *γ*_r_ and *γ*_a_ decrease continuously with similar trends, while the loss ratio *f* remains nearly unchanged, indicating a stable loss balance and absorption response (Figure 2k). Using this method, pixelated metasensors with specific loss rates can be constructed. We fabricated the corresponding samples. The experimentally measured absorption spectra together with the corresponding TCMT fitting results are presented in Figure 2l. The radiative and intrinsic loss rates extracted from the fitting are summarized in Figure 2m. Consistent with the numerical simulations, both loss rates exhibit only minor variations among the different devices, indicating that the loss channels remain nearly unchanged despite the resonance‐frequency tuning. The phase spectra are shown in Figure 2n. The phase evolution around the resonance is nearly identical for all devices, further verifying the stable regulation of the coupling condition and demonstrating that the resonance frequency can be tuned with minimal perturbation to the loss rates. Besides the three structural parameters discussed above, the resonator width *w* also affects the loss and the resonance frequency (Figure S4). Since fully decoupled control of the resonance frequency and loss is already achieved with these three parameters, *w* is not considered further. All of the resonance parameters extracted by TCMT fitting in this section can be found in Tables S1–S5.

### 2.3 Loss‐Engineered Sensing Optimization Strategy

For a resonator‐molecule system, the magnitude of the optimal loss rate depends on the coupling strength between them and on the absorption characteristics of the molecular vibrational modes. Therefore, the resonator and the molecule should be analyzed as an integrated whole. Figure 3a describes the behavior of the resonator‐molecule coupling system under the excitation of THz waves. Here, the resonator *P* acts as a bright mode that is directly excited by the incident THz wave. In contrast, the molecular vibration mode *M*, acting as a dark mode, cannot directly interact with external THz waves, making it difficult to observe directly in the far‐field response. When molecules are loaded onto the metasensor, near‐field coupling excites the dark mode indirectly with a coupling strength *µ*. The vibrational spectrum of the molecules can then be detected in the far field through its modulation of the resonator spectrum. The metasensor therefore acts as the bridge that enhances the molecular vibrational response. The absorption response of the coupling system can be calculated by (see Section S2 for the detailed TCMT derivation):

(3) $$A=1-|\frac{2\gamma_{r}}{j\left(\omega -\omega_{0}\right)+\left(\gamma_{r}+\gamma_{a}\right)+\left(\frac{\mu^{2}}{j\left(\omega -\omega_{m}\right)+\gamma_{m}}\right)}-1{|}^{2}$$

where *ω*_m_ is the vibrational frequency of the molecules, *γ*_m_ is the absorption loss of the vibrational mode, and µ is the coupling strength between the resonator and molecules. The enhancement amplitude of molecular vibrational signals can be calculated as:

(4) $$\Delta A=A-A{|}_{\mu =0}$$

**FIGURE 3:**
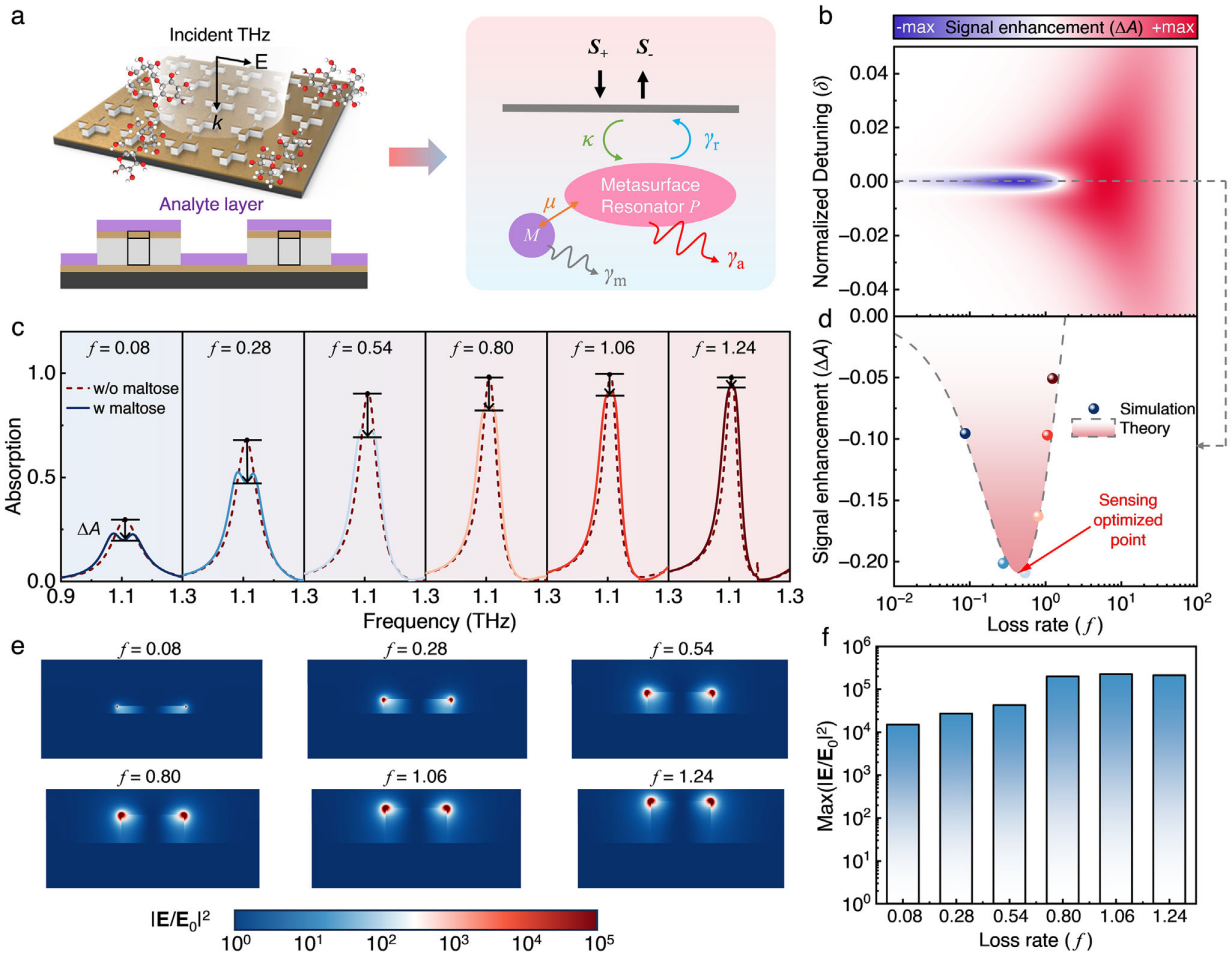
Loss‐engineering sensing optimization strategy. (a) Schematic of the meta‐pixel and analyte coupling system and its TCMT model. (b) Molecular signal enhancement as a function of normalized detuning *δ* and loss rate *f*. (c) Simulated absorption spectra of the metasurfaces with/without a 2‐µm‐thick maltose layer at different loss rate *f*. (d) Theoretical and simulated signal enhancement (Δ*A*) of molecular signals as a function of loss rate *f*. (e) Electric field enhancement (|**E**/**E**_0_|^2^) distribution and (F) maximum electric field enhancement (max(|**E**/**E**_0_|^2^)) of the meta‐pixel with different loss rates at the resonance frequency.

Based on Equations (3) and (4), Figure 3b shows the theoretical enhancement amplitude as a function of the loss rate *f* and the normalized detuning *δ* (defined as *δ* = (*ω*_0_ – *ω*_m_) / *ω*_m_). The enhanced molecular signals fall into two regions: negative modulation in the UC regime and positive modulation in the OC regime, with comparable maximum amplitudes. For zero detuning, the loss rate that offers the highest molecular fingerprint signal enhancement can be identified (gray dashed line in Figure 3b). The molecular fingerprint signal enhancement can also be obtained analytically (see Section S2 for the detailed derivation):

(5)

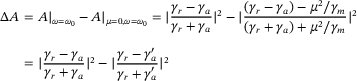

where *γ*′_a_ = *γ*_a_ + *µ* ^2^/*γ_m_*. In Equation (5), the molecules act as an additional intrinsic loss channel (described by *µ* ^2^/*γ*_m_), resulting in the resonance absorption shift to the left along the curve in Figure 2b, and the corresponding absorption modulation can be calculated using Equation (5). The modulation that the analyte imposes on the absorption of the metasensor is therefore governed by the slope of the absorption curve, d*A*/d*f*. Thus, near the critical‐coupling point (*f* = 1), the curve reaches a maximum and its slope is minimal, as shown in Figure 2b, whereas in the under‐coupled regime (*f* < 1), the slope d*A*/d*f* is substantially larger. Therefore, the system exhibits enhanced sensitivity to loss perturbations in the UC regime, even though the local field intensity is not maximized.

We then validate the above theoretical results through simulation. Maltose is chosen as the target molecule because it has a vibrational fingerprint at 1.11 THz. This peak originates predominantly from intermolecular interactions and from rotation of the −CH_2_OH group, the six‐membered ring, and the −OH group [63]. The permittivity of maltose is described by a Lorentz model ^[^ ^4^ ^]^:

(6) $$\varepsilon \left(\omega \right)=\varepsilon_{\infty }+\sum_{n}\frac{S_{i}^{2}}{{\omega_{i0}}^{2}-\omega^{2}-i\gamma_{i}\omega }$$

where *ε*_∞_ is the background permittivity, ω*_i_*_0_ is the vibrational frequency, *S_i_* and *γ_i_* are related to the vibrational strength and linewidth. Equation (6) describes the dielectric constant of a substance with *i*‐order fingerprint vibration peaks, and the parameters are obtained by fitting the measured absorption peaks, where *ε*_∞_ = 3.06, *ω_i_*_0_ = 1.11 THz, *S_i_* = 0.28, *γ_i_* = 0.06 THz. Next, metasensors with different loss rates are constructed and their resonant frequencies are designed to match the vibrational fingerprint of the maltose at 1.11 THz. By covering the metasensors with the maltose layer, the absorption spectra of the coupling system are obtained (Figure 3c). Compared with the bare metasensors, those coated with a maltose layer show clear dips (quantified by Δ*A*) at the molecular vibration frequency. The electromagnetically induced transparency (EIT) phenomenon arises from the near‐field coupling between the molecule and the resonator. The extracted molecular signal enhancement (Δ*A*) agrees well with the theoretical values calculated by Equation (5), as shown in Figure 3d. In Figure 3d, as the loss rate *f* increases, Δ*A* first gradually increases and then decreases, reaching a maximum value near *f* = 0.5, corresponding to an absorption of about 0.9 according to Equation (1).

Figure 3e,f shows the electric field intensity (|**E**/**E**_0_|^2^) distribution and maximum electric field enhancement of the meta‐pixel with different loss rates at resonance frequency. The field intensity reaches its maximum near the CC point (*f* ≈ 1), whereas the highest sensitivity appears in the UC region in Figure 3d. This apparent discrepancy arises because molecular fingerprint sensing is governed not solely by the local field intensity but by the change in absorption induced by the analyte interacting with the resonator's loss channels. In the UC regime, the system is more sensitive to perturbations in absorption loss introduced by molecular vibrations, producing a larger modulation of the absorption spectrum even when the near‐field intensity is not at its peak. At critical coupling, by contrast, the resonator is optimized for energy storage, and the response to small changes in loss is weaker than in the UC region. This behavior highlights the essential role of loss engineering in optimizing molecular fingerprint sensing.

### 2.4 Metasensor for Molecular Vibrational Fingerprint Retrieval

Guided by the above theoretical analysis, pixelated metasensors can be designed for molecular‐vibration fingerprint sensing. The loss rate of the proposed metasensor is tuned mainly through the out‐of‐plane height of the resonators, which makes it difficult to prepare by conventional lithography‐based 2D fabrication. Here, we employ projection micro‐stereolithography (PµSL) 3D printing (nanoArch S130, BMF Material Technology Inc.) to fabricate the designed metasensors in three steps (Figure 4a). First, a layer of optically thick copper is deposited on a cleaned silicon wafer using electron beam (EB) deposition to prevent transmission. Second, the dielectric layer is directly printed onto the copper‐plated silicon wafer in a layer‐by‐layer manner using the PµSL‐based 3D printing technology. Finally, a gold layer with a thickness of 200 nm is deposited on the printed dielectric layer using the EB deposition method. Figure 4b shows optical images of the fabricated metasensor with a 3 × 3‐pixel array, in which the pixels are numbered P1 to P9. The entire metasensor measures 20 × 20 mm, and each pixel is smaller than 5 × 5 mm (about 20 × 20 resonant wavelengths). Figure 4c shows the surface morphology of pixel P5 measured with a 3D surface metrology microscope (DCM8, Leica), confirming high structural uniformity.

**FIGURE 4:**
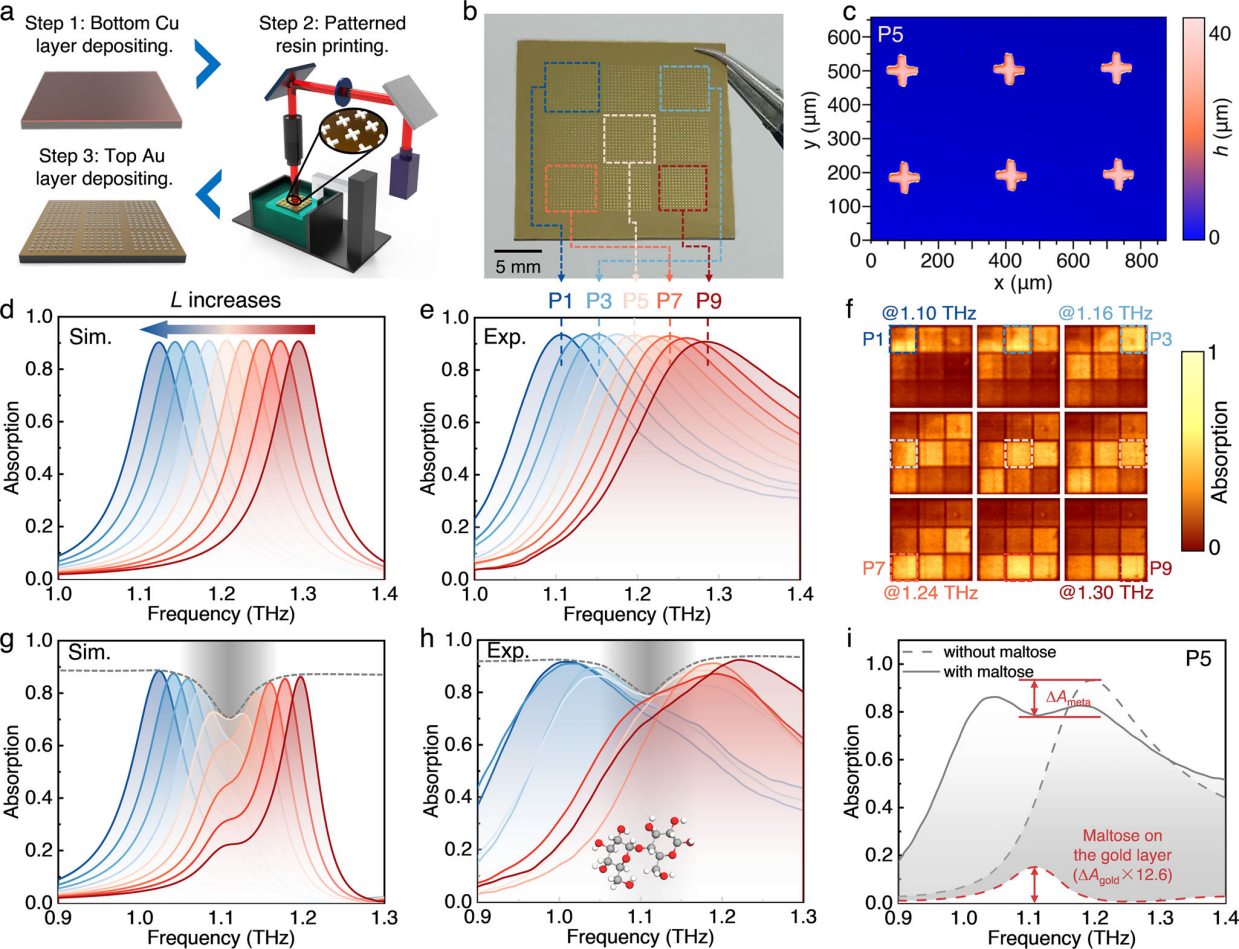
Metasensor for maltose fingerprint retrieval. (a) Schematic of the 3D printing technique‐based three‐step fabrication strategy. (b) The optical image of the fabricated nine‐pixel metasensor. (c) Laser scanning confocal microscope images of Pixel 5. (d) Simulated absorption spectra of the nine meta‐pixels. (e) Measured absorption spectra of the fabricated metasensor. (f) THz absorption images of the metasensor at the resonant frequencies of each pixel. (g) Simulated fingerprint retrieval of maltose. (h) Measured fingerprint retrieval of maltose using the fabricated metasensor. (i) The absorption spectra of pixel 5 (P5) with and without maltose. The red dashed line at the bottom shows the maltose signal on the gold layer (Multiplied by 12.6 times).

The simulated and measured absorption spectra of the nine pixels are shown in Figure 4d,e. The reconstruction accuracy of the proposed pixelated metasensor is mainly determined by the resonance frequency spacing between adjacent pixels, which was designed to be approximately 0.025 THz in this work to enable reliable reconstruction of typical THz molecular fingerprints while remaining compatible with the fabrication resolution and printable area constraints of the employed microscale 3D‐printing system. Notably, resonances are intentionally designed above the molecular vibrational frequency to mitigate resonance shifts induced by the real part of the molecular refractive index (see Figure S5). The absorption amplitude is stable at around 0.9, which corresponds to the optimized loss rate as theoretically demonstrated in the previous section. Differences in the resonance linewidths between the simulated and experimental spectra can be attributed to the surface roughness introduced during 3D printing, which increases the radiative loss *γ*_r_ and may add intrinsic loss *γ*_a_. The resonance linewidth is broadened as a result, and the loss rate changes slightly. Figure 4f shows the absorption images of the metasensor at the resonant frequencies of each pixel. The pixels light up in sequence as the imaging frequency matches the resonance frequency of each pixel. Because different pixels reach maximum absorption at different resonant frequencies, fingerprint enhancement occurs across the whole design band.

To assess the role of the metasensor in molecular fingerprint enhancement, we use it to reconstruct the fingerprint of maltose. Figure 4g,h shows the simulated and measured fingerprint absorption spectra of a 5‐µm‐thick maltose layer retrieved with the metasensor. In the experiments, approximately 20 µL of a 150 mg/mL maltose aqueous solution was deposited onto the metasensor chip, resulting in an average effective maltose layer with an estimated thickness of approximately 5 µm after solvent evaporation. The absorption spectrum of each pixel shows a collective dip at 1.11 THz, set by the intrinsic vibrational absorption of maltose. By extracting the envelope of each pixel's spectrum, the reconstructed fingerprint spectrum of maltose can be obtained (shown by the gray dashed line in Figure 4g,h). Specifically, the reconstructed fingerprint is obtained by fitting a Lorentzian function to the collective spectral responses of all pixels, such that the fitted curve captures the consistent molecular‐induced modulation across the dataset. This effectively forms a smooth envelope constrained by the multi‐pixel measurements, rather than relying on individual extrema. The deepest dip occurs for P5, whose resonance lies at 1.11 THz, indicating the strongest coupling between the metasensor resonance and the molecular vibration mode (Figure 4i). For comparison, the red dashed curve at the bottom of Figure 4i shows the absorption spectrum of maltose on a gold mirror, scaled by a factor of 12.6 to match the absorption change induced by maltose at the resonance of P5. The enhancement factor can be defined as the ratio between the absorption change at the P5 resonance Δ*A*_meta_, and that measured for the same analyte on the gold mirror Δ*A*_gold_, and can be calculated as: Δ*A*_meta_/Δ*A*_gold_ = 12.6. Table S7 shows a comprehensive performance comparison of this work with recent THz fingerprint metasensor studies. Overall, by jointly optimizing loss and aligning the resonant frequency, a pronounced enhancement of the molecular fingerprint spectra is achieved. Although the demonstrated design spans only about 0.2 THz, this limitation arises primarily from the printable area and fabrication resolution of the 3D‐printing system used here. The proposed design strategy is not fundamentally limited to a narrow operational bandwidth. Through the co‐optimization of the three structural parameters *p*_0_, *L*_0_, and *S*, broadband fingerprint‐enhanced sensing covering the 0.5–2.5 THz range can in principle be achieved using Equation (2), as demonstrated by the numerical results provided in Figure S6. Therefore, the proposed loss‐engineered THz metasensor design method holds considerable potential for mixture identification and quantitative sensing applications requiring broadband molecular fingerprint analysis.

### 2.5 Metasensor for Molecular Vibrational Strong Coupling

Next, the potential of the proposed metasensor for realizing strong coupling with molecular vibrations is investigated. Strong coupling occurs when the rate of energy exchange between light and molecular vibrations exceeds their respective loss rates, resulting in the formation of hybrid light‐matter states known as polaritons [64, 65, 66]. Owing to its optimized coupling strength and tunable coupling regime, the proposed pixelated metasensor is well suited to reaching this regime, since the multi‐pixel design gives precise control over the detuning between photonic resonances and molecular vibrations through unit‐cell scaling [67], while its optimized coupling strength facilitates efficient energy exchange. As illustrated in Figure 5a, strong coupling can be understood as the interaction between two resonant modes. When the metasensor resonance, seen as the photon mode, approaches the vibrational mode of the analyte, the interaction between them leads to an anti‐crossing behavior characterized by a Rabi splitting and two hybrid energy branches: the upper polariton branch (UPB) and the lower polariton branch (LPB) [68]. This spectral splitting is widely recognized as a hallmark of strong light‐matter coupling, and the magnitude of the Rabi splitting serves as a direct measure of the coupling strength.

**FIGURE 5:**
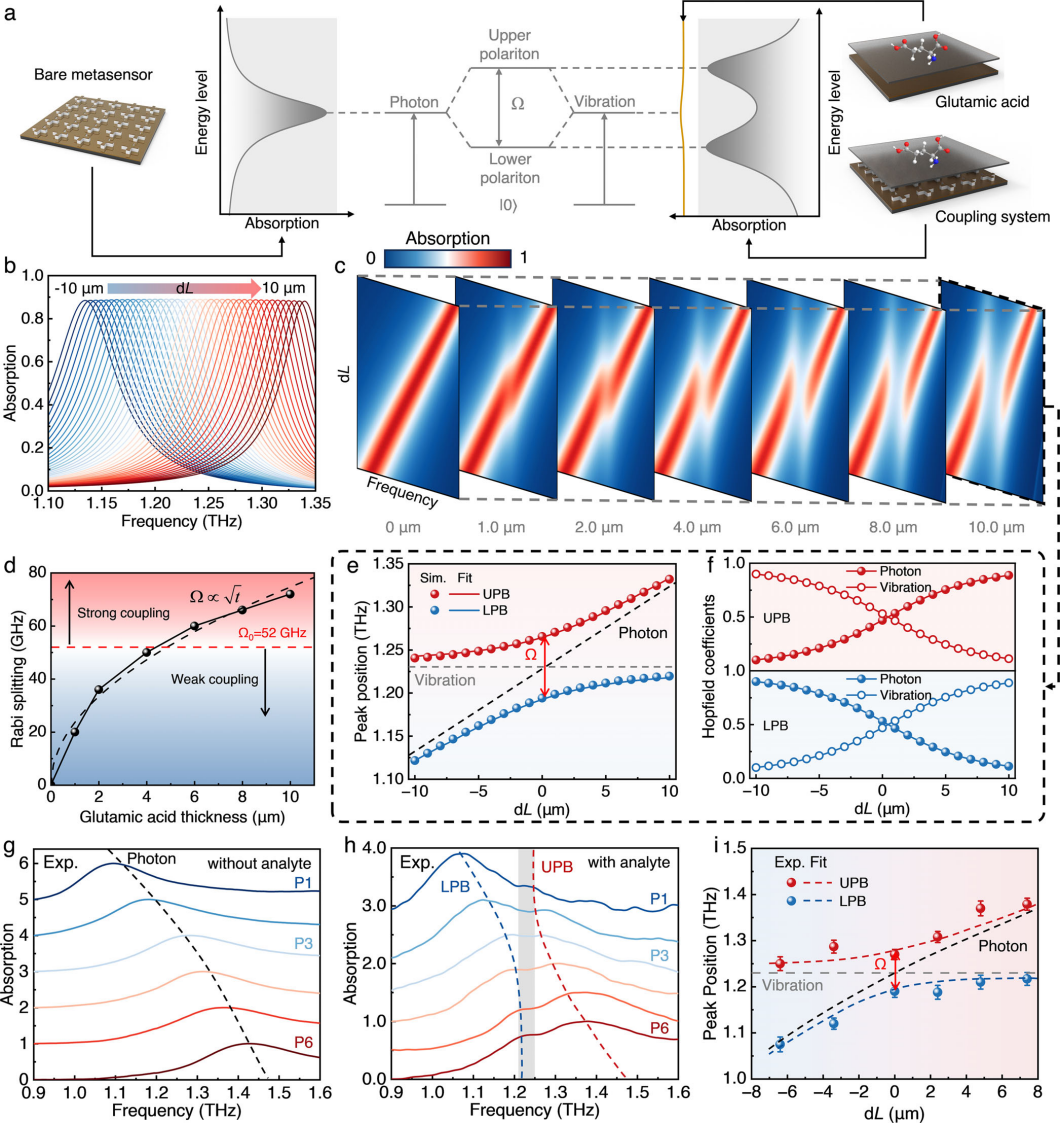
Vibrational strong coupling between the metasensor and glutamic acid molecular vibrations. (a) Schematic of molecular vibrational strong coupling. (b) Simulated absorption spectra of the metasensor with different geometrical detuning parameters d*L*. (c) Absorption maps of the coupling system with different thickness of glutamic acid. (d) Rabi splitting Ω of the coupling system for different glutamic acid layer thicknesses, where the red dashed line indicates the strong coupling limit. (e) Calculated dispersion curves of the lower polariton branch (LPB) and upper polariton branch (UPB) of the coupling system. The black and gray dashed lines represent the photon and the vibration mode, respectively, and the red solid arrow denotes the Rabi splitting Ω. (f) Calculated Hopfield coefficients of UPB and LPB, respectively, which show the weights of photon and vibration modes in the two branches. (g) Measured absorption spectra of the fabricated metasensor. (h) Measured absorption spectra of the metasensor covered with a 10‐µm‐thick glutamic acid layer. (i) Extracted dispersion curves of LPB and UPB of the coupling system.

The vibrational band of glutamic acid at 1.23 THz is selected to investigate strong coupling. To maximize the coupling strength between the metasensor and the glutamic acid molecules, we optimized the loss rate *f* of the metasensor for the absorption characteristics of glutamic acid at 1.23 THz (see Figure S7). We then constructed a series of resonances to provide broadband spectral overlap (Figure 5b). Subsequently, by loading glutamic acid layers with varying thickness onto the metasensor, the coupled system transitions from weak coupling to strong coupling (Figure 5c). When the thickness of the glutamic acid molecular layer is small, for example 1 µm, the vibrational fingerprint can be reconstructed from the envelope of all pixel spectra (Figure S8), but no anti‐crossing is visible in the intensity spectra (Figure 5c), indicating that the system has not yet entered the strong coupling regime. As the thickness of the glutamate layer increases beyond 4 µm, spectral splitting appears at the molecular vibration frequency (Figure 5c). Based on coupled‐oscillator theory, the upper and lower polariton branches (UPB and LPB) formed by the splitting can be written as (see Section S10):

(7) $$\omega_{\pm }=\frac{\omega_{ph}+\omega_{\mathrm{vib}}}{2}\pm \frac{1}{2}\sqrt{4g^{2}+{\left(\omega_{ph}-\omega_{\mathrm{vib}}\right)}^{2}}$$

where *ω*_ph_ and *ω*_vib_ denote the frequencies of the metasensor resonance and the molecular vibration mode, respectively, and *g* represents the coupling strength between the two modes. Using Equation (7), we fitted the spectra of the coupling system and obtained the Rabi splitting Ω at different thicknesses (Figure 5d). Strong coupling occurs when the Rabi splitting Ω satisfies

(8) $$\Omega >\frac{\gamma_{ph}+\gamma_{m}}{2}$$

where *γ*_ph_ and *γ*_m_ represent the linewidths of the pixel resonance and the vibrational fingerprint of glutamic acid, respectively. In experiments, *γ*_ph_ = 64 GHz and *γ*_m_ = 40 GHz, and the value of Ω can be calculated as 52 GHz. Thus, the transition from weak to strong coupling regimes occurs when the glutamic acid film thickness exceeds 4 µm (Figure 5d). The extracted Rabi splitting Ω is fitted to a square root function of the glutamic acid thickness *t*, which is characteristic of strong coupling [67]. The mode volume of the localized metasensor resonance is calculated as *V*_m_ = ∫ε(**r**)|*E*(**r**)|^2^d^3^ *V* / max(ε(**r**)|*E*(**r**)|^2^) = 16.9 µm^3^, corresponding to a normalized volume *V*_m_/λ^3^ = 1.16×10^−6^. This small normalized volume indicates a tightly confined optical mode. Combined with the observed Ω ∝ *t* ^0.5^ scaling of the Rabi splitting and the linewidth criterion Ω > (*γ*_ph_+*γ*_m_)/2, these results confirm that the observed anti‐crossing originates from genuine vibrational strong coupling. Loss engineering is central to optimizing light‐matter interaction in the strong‐coupling regime. Tailoring the photonic decay rates balances radiative and intrinsic dissipation, which makes energy exchange between the metasensor resonance and the molecular vibration more efficient and the strong‐coupling criterion easier to satisfy. Besides, we extracted the molecular enhancement amplitudes for different analyte‐layer thicknesses and compared the three‐dimensional out‐of‐plane configuration with its two‐dimensional in‐plane counterpart (Figure S9). The results reveal a clear monotonic dependence of the reconstructed spectral enhancement on molecular thickness (Figure S10), demonstrating the potential of the proposed metasensor for quantitative molecular detection in addition to qualitative fingerprint identification. More importantly, while the response of the in‐plane configuration gradually approaches saturation with increasing molecular thickness, the out‐of‐plane configuration maintains a pronounced thickness‐dependent increase and yields substantially larger enhancement at higher molecular loadings (Figure S10). This extended dynamic response demonstrates the improved sensing sensitivity enabled by the three‐dimensional loss‐engineered architecture.

The dispersion curves of the coupling system with a 10‐µm‐thick glutamic acid layer are mapped with Equation (7), which relates the frequencies of the upper and lower polaritons to the detuning d*L* (Figure 5e). The dispersion curves reveal the formation of a characteristic polariton branch near the absorption frequency of 1.23 THz, which is the collective vibration frequency of the glutamic acid molecules. Furthermore, to better understand the strong coupling process, the weights of photon and vibration modes in the UPB and LPB are extracted, which are related to the Hopfield coefficients *a*_ph_ and *a*_vib_ (see Section S10), as shown in Figure 5f. In the case of a smaller d*L*, the upper polariton branch is mainly composed of vibration modes. As d*L* increases, the Hopfield coefficient of the vibration mode decreases while the photon mode increases. When d*L* reaches approximately 0.5 µm, the photonic and vibrational Hopfield coefficients become nearly equal, indicating an approximately zero‐detuning condition and maximal mode hybridization within the strong‐coupling regime. The Hopfield coefficients show the opposite trend for the LPB. Consequently, the Hopfield coefficient distributions illustrate the intermixing behavior between the photon and molecular vibration modes, and provide evidence for the formation of strong mode coupling.

Figure 5g,h presents the measured absorption spectra of the metasensor (P1–P6) without and with a 10‐µm‐thick glutamic acid layer, respectively. In the absence of the analyte, each pixel exhibits a distinct optical resonance corresponding to its designed frequency (Figure 5g). Upon the introduction of glutamic acid, these discrete resonances vanish and are replaced by two new hybridized branches, the upper and lower polariton modes, accompanied by a pronounced mode splitting (Figure 5h). This spectral evolution signifies the transition from weak to strong coupling between the photonic resonances of the metasensor and the vibrational mode of glutamic acid, where the coherent exchange of energy dominates over the loss channels. Figure 5i summarizes the extracted positions of the upper and lower polariton branches, clearly revealing an anti‐crossing behavior with a Rabi splitting of approximately 83 GHz. Such a sizable and spectrally resolved splitting provides compelling evidence of vibrational strong coupling. This observation aligns well with the simulated results in Figure 5e, validating the effectiveness of loss‐engineered metasensors in achieving and controlling strong light‐matter interactions in the THz regime.

### 2.6 Discussion

Spectral overlap and coupling strength are two fundamental factors governing the performance of vibrational fingerprint sensing. However, research efforts in the THz domain have so far primarily concentrated on extending the resonance coverage of metasurfaces through multi‐resonant or broadband designs, aiming to capture multiple vibrational fingerprints simultaneously. While these approaches effectively address the challenge of spectral detuning, the coupling strength in the THz regime has received little systematic attention (Table S7). Although coupling can be increased to some extent through functionalization or nanoscale gap design, these routes add cost and complexity. In this context, loss engineering, which governs the balance between radiative and intrinsic dissipation, provides an alternative and more intrinsic approach to enhance coupling strength and overall sensing performance.

Our strategy optimizes metasensor sensitivity through loss engineering and achieves continuous tuning of the loss rate by varying the dielectric layer height of the meta‐atoms. This out‐of‐plane degree of freedom provides an effective handle to adjust the balance between radiative and intrinsic losses, thereby enabling optimization of the sensing performance for THz molecular fingerprint enhancement. Moreover, the adoption of the 3D printing‐based fabrication method overcomes the inherent limitations of conventional planar lithography, which lacks the flexibility to precisely realize out‐of‐plane structural parameters. The device demonstrated here consists of nine pixels, a number set by the printable area of the 3D printing system. Looking forward, the use of advanced large‐area micro‐nanoscale printing technologies could enable devices with higher pixel densities, expanded frequency coverage, and finer frequency step sizes.

## 3 Conclusion

In summary, we have proposed and experimentally demonstrated a loss‐engineered pixelated metasensor for THz molecular vibrational fingerprint enhancement. Different from conventional metasensor designs that primarily focus on maximizing local field enhancement, the present work establishes loss engineering as a general strategy for tailoring dissipative light‐matter interaction through controlled radiative and intrinsic losses. Using TCMT, we identified the loss rate as the key factor governing sensing performance. We introduce the out‐of‐plane resonator height as an effective tuning parameter that enables continuous control of the loss rate, thereby allowing systematic coupling optimization of the metasensing system. Furthermore, the proposed pixelated metasensor with an out‐of‐plane configuration can be readily fabricated by leveraging 3D printing technology. The proposed framework enables enhanced molecular fingerprint sensing, broadband pixelated spectral reconstruction, and strong light‐matter coupling with molecular vibrations. Overall, the proposed sensing architecture offers an effective route to high‐performance, high‐throughput, and low‐cost biomedical sensing.

## 4 Experimental Section/Methods

### 4.1 Numerical Simulation

Electromagnetic simulations were performed with the commercial finite‐element solver CST Studio Suite. The source was set as an incident plane wave, and copper and gold were described by the built‐in material library parameters. The permittivity of the resin was set to 2.755+0.41j at 1 THz, with the loss component effectively adjusted from the measured material value to account for additional fabrication‐induced and scattering losses observed experimentally. Adaptive mesh refinement and periodic boundary conditions were used in the frequency‐domain solver, and the electric field distributions were calculated with E‐field monitors at the target frequencies.

### 4.2 Sample Preparation

Projection micro‐stereolithography (PµSL) forms objects layer by layer, allowing the creation of structures with out‐of‐plane features at the micron scale, such as high aspect ratio pillars, inclined surfaces, gradient surfaces, and spiral structures. Before printing, a digital model of the dielectric layer structure was constructed in computer‐aided design (CAD) software, and slicing software generated the pattern for each printed layer. During printing, each pattern serves as a light mask that projects ultraviolet light onto the liquid resin and cures it into the desired shape. After each layer is cured, the stage moves down so that fresh resin covers the cured structure, and exposure and curing repeat until the entire model is printed (Figure S11). The printed model was then cleaned with ethanol and post‐cured in an ultraviolet box for 15 min to further enhance structural stability.

### 4.3 Terahertz Spectroscopy Measurement

All absorption spectra were measured using a commercial THz time‐domain spectroscopy (THz‐TDS) system. The optical path of the THz‐TDS system is shown in Figure S12. During the measurement, the THz beam was focused onto the sample surface, and the reflected THz signal was routed to the photodetector to capture the far‐field spectral response. The signal reflected from a gold mirror was first recorded as a reference, and all other spectra were normalized to it through: *R* = (*E*_dev_ / *E*_ref_)^2^, where *E*_dev_ and *E*_ref_ represent the measured electric field intensity of the device and the reference, respectively. All data were averaged over 100 repeated measurements to ensure reliability and stability. Because the device carries a bottom metal reflector, the absorption spectra follow as: *A* = 1 – *R*.

The THz absorption images were obtained by raster scanning the integrated transmitting and receiving probe on the *x*–*y* motion stage of the THz‐TDS system. The scanning step for each image was 0.2 mm, and the scanning range 16 × 16 mm, which covers all of the pixels.

## Author Contributions

**Chenglin Yang**: software, methodology, conceptualization, writing – original draft, visualization, formal analysis, investigation, validation, data curation. **Yuqing Cui**: methodology, formal analysis, software, data curation, investigation. **Donghai Han**: methodology, software, formal analysis. **Yushan Hou**: methodology, software, formal analysis. **Zhonglei Shen**: methodology, writing – review and editing, supervision, conceptualization. **Andreas Tittl**: funding acquisition, writing – review and editing, supervision, methodology, conceptualization. **Liuyang Zhang**: conceptualization, methodology, writing – review and editing, supervision, funding acquisition, resources, project administration. **Ruqiang Yan**: supervision, writing – review and editing. **Xuefeng Chen**: writing – review and editing, supervision.

## Funding

National Natural Science Foundation of China, Grant 52175115, Grant 92360306; Fundamental Research Funds for the Central Universities, Grant. 11913224000045; Deutsche Forschungsgemeinschaft (German Research Foundation) under grant numbers EXC 2089/1–390776260 (Germany's Excellence Strategy) and TI 1063/1 (Emmy Noether Program), and by the European Union (ERC, METANEXT, 101078018).

## Conflicts of Interest

The authors declare no conflicts of interest.

## Supporting information




**Supporting File**: advs78233‐sup‐0001‐SuppMat.docx.

## Data Availability

The data that support the findings of this study are available from the corresponding author upon reasonable request.
