# Supplementary material for: Loss‐Engineered Terahertz Pixelated Metasensors for Enhanced Molecular Vibrational Fingerprint Sensing
Source: Adv Sci (Weinh). 2026 Oct 14:e78233. Online ahead of print. doi: 10.1002/advs.78233 (PMC13669216; doi:10.1002/advs.78233)
Supplement: Supplementary file 1 [file ADVS-9999-e78233-s001.docx]

Supporting Information

Loss-engineered Terahertz Pixelated Metasensors for Enhanced Molecular Vibrational Fingerprint Sensing

Chenglin Yang, Yuqing Cui, Donghai Han, Yushan Hou, Zhonglei Shen*, Andreas Tittl*, Liuyang Zhang*, Ruqiang Yan, Xuefeng Chen

**The supporting information includes:**

[S1.	Resonance property of the cross-shaped meta-atom.	3](#_Toc240889088)

[S2.	Temporal coupled model theory (TCMT) analysis for metasurface and the molecule-metasurface coupling system.	4](#_Toc240889089)

[S3.	The absorption spectra and TCMT fitting results of meta-pixels with different geometric parameters.	7](#_Toc240889090)

[S4.	The effect of the resonator width *w* on losses.	8](#_Toc240889091)

[S5.	Resonance parameters extracted by TCMT fitting.	9](#_Toc240889092)

[S6.	The effects of real and imaginary parts of the refractive index of the analyte on the metasensor resonance.	11](#_Toc240889093)

[S7.	Ultra-broadband pixelated metasensor design.	12](#_Toc240889094)

[S8.	Optimization of the metasensor for glutamic acid vibrational enhancement.	13](#_Toc240889095)

[S9.	Absorption spectra of the metasensor and its 2D counterpart with different thickness of the glutamic acid layer coating.	14](#_Toc240889096)

[S10.	Strong coupling system analysis.	15](#_Toc240889097)

[S11.	Comparison of different metasensors for molecular vibrational sensing.	17](#_Toc240889098)

[S12.	Schematic diagram of PμSL-based 3D printing technique.	19](#_Toc240889099)

[S13.	Optical path of the THz-TDS.	20](#_Toc240889100)

S1. Resonance property of the cross-shaped meta-atom.

**Figure S1 Resonance property of the meta-atom.** (a) Electric field enhancement in the meta-atom for TE and TM linearly polarized excitation at the resonant wavelengths. (b) The electric field enhancement along the white dashed line in (a). (c) Multipole expansion analysis. Here, electromagnetic multipole expansion is utilized to evaluate each multipole and their contributions to the far-field radiation. In the Cartesian coordinate system, the five leading multipole moments, including the electric dipole moment ***P***, toroidal dipole moment ***T***, magnetic dipole moment ***M***, electric quadrupole moment ***Q*_e_** and magnetic quadrupole moment ***Q*_m_** are considered^[1]^.

S2. Temporal coupled model theory (TCMT) analysis for metasurface and the molecule-metasurface coupling system.

**Figure S2 Schematic of the TCMT model of the molecule-metasurface coupling system.**

The TCMT model for the one-port single-mode system, as shown in Figure S2a which can be described as follows:^[2,3]^

where *P* is the mode amplitude of the metasurface, *ω*_0_ is the resonance frequency of the metasurface. For time-harmonic field, where d*A*/d*t*=j*ωt*, therefore, the absorption can be expressed by

$$[j(\omega-\omega_{0})+\gamma_{r}+\gamma_{a}]A_{0}=\kappa S_{+}$$

*κ* is related to *γ*_r_ and be expressed as follows

$$\kappa=\sqrt{2\gamma_{r}}$$

so, the reflectance *r* and absorption *A*_0_ can be expressed as

The resonance absorption can be obtained when *ω*=*ω*_0_

$$A_{\max}=\frac{4\gamma_{r}\gamma_{a}}{(\omega_{0}-\omega_{0})^{2}+(\gamma_{r}+\gamma_{a})^{2}}=\frac{4\gamma_{r}\gamma_{a}}{(\gamma_{r}+\gamma_{a})^{2}}=\frac{4f}{(1+f)^{2}}$$

where *f* is defined as the ratio of radiative loss to intrinsic loss (*f*=*γ*_r_/*γ*_a_). The profile of Equation (S8) is plotted in Figure 2b of the main text. It deserves noting that the perfect absorption can be achieved at the critical coupled state, i.e., *γ*_r_=*γ*_a_. Since *A*_0_ =2*A*_h_, the full width at half maximum can be gained by:

$$\Gamma^{FWHM}=2|\omega_{1}-\omega_{0}|=2(\gamma_{r}+\gamma_{a})$$

and the total *Q*-factor is gained by

$$Q=\frac{\omega_{0}}{\Gamma^{FWHM}}=\frac{\omega_{0}}{2(\gamma_{r}+\gamma_{a})}$$

According to the definition of radiative and intrinsic *Q*-factors, i.e., *Q*_r_ = *ω*_0_/2*γ*_r_ and *Q*_a_ = *ω*_0_/2*γ*_a_, and the relationship among these three *Q*-factors can be described as

$$Q=\frac{Q_{r}Q_{a}}{Q_{r}+Q_{a}}$$

The abrupt phase variation Δ*Φ* at *ω*_0_ can be derived from the Equation (S6) as

$$\Delta\Phi=angle(\frac{j\omega_{0-}+\gamma_{r}-\gamma_{a}}{-j\omega_{0-}+\gamma_{r}+\gamma_{a}})-angle(\frac{j\omega_{0+}+\gamma_{r}-\gamma_{a}}{-j\omega_{0+}+\gamma_{r}+\gamma_{a}})$$

where *ω*_0-_ (*ω*_0+_) denotes the left-hand (right-hand) limit of *ω*_0_. For the critical coupled state (*γ*_r_ = *γ*_a_), the phase variation Δ*Φ* = *π*. But for general cases, two coupled regions, i.e., (1) the under coupled region and (2) the over coupled region are defined according to the Equation (S12).

(1) Under-coupled region

In the under-coupled region (*γ*_r_ < *γ*_a_, Δ*Φ* = 0~π), the reflection *R*_0_ at the resonant frequency can be calculated as

$$\sqrt{R_{0}}=-\frac{\gamma_{r}-\gamma_{a}}{\gamma_{r}+\gamma_{a}}$$

so, the radiative *Q*-factor *Q*_r_ and intrinsic *Q*-factor *Q*_a_ can be obtained as:

(2) Over-coupled region

In the under-coupled region (*γ*_r_ > *γ*_a_, Δ*Φ* = 2π), the reflection *R*_0_ at the resonant frequency can be calculated as

$$\sqrt{R_{0}}=\frac{\gamma_{r}-\gamma_{a}}{\gamma_{r}+\gamma_{a}}$$

so, the radiative *Q*-factor *Q*_r_ and intrinsic *Q*-factor *Q*_a_ can be obtained as:

In practice, we need to judge the state of the system according to phase variation *Δ*Φ first, and then the total *Q*-factors can be extracted from the absorption spectra by the Equation (S10), while the radiative *Q*-factor *Q*_r_ (or intrinsic *Q*-factor *Q*_a_) can be extracted by the Equation (S14) and (S17) (or Equation (S15) and (S18)). Finally, the radiative/intrinsic loss can be calculated based on the definition of the *Q*_r_ and *Q*_a_.

Figure S2b shows the TCMT model for the molecule coupled to the metasurface with a coupling coefficient *μ*. Then, the coupling system can be described as

where *M*, *ω*_m_, and *γ*_m_ represent the amplitude, angular frequency, and absorption loss of the molecular vibration, respectively. Similarly, for time-harmonic field, d*M*/d*t*=j*ωt*. Based on Equation (S19) - (S22), we can obtain the absorption after coupling

$$A=1-|\frac{\kappa^{2}}{j(\omega-\omega_{0})+(\gamma_{r}+\gamma_{a})+(\frac{\mu^{2}}{j(\omega-\omega_{m})+\gamma_{m}})}-1|^{2}$$

When *μ* = 0, the absorption of the bare metasurface can be described as

$$A_{0}=1-|\frac{\kappa^{2}}{j(\omega-\omega_{0})+(\gamma_{r}+\gamma_{a})}-1|^{2}$$

The enhancement of the vibrational signal *ΔA* can be expressed as a difference in absorption with and without molecule

$$\Delta A=A-A_{0}$$

Based on Equations (S23) - (S25), we can obtain the theoretical enhancement magnitude of the molecular vibration signal as a function of the loss rate *f* and the normalized detuning (*δ*=(*ω*_0_-*ω*_m_)/*ω*_m_), which is shown in Figure 3(b) of the main text. With the zero detuning, the molecular fingerprint signal enhancement can also be obtained as:

$$\begin{matrix} \Delta A=A|_{\omega=\omega_{0}}-A|_{\mu=0,\omega=\omega_{0}}=|\frac{\gamma_{r}-\gamma_{a}}{\gamma_{r}+\gamma_{a}}|^{2}-|\frac{(\gamma_{r}-\gamma_{a})-\mu^{2}/\gamma_{m}}{(\gamma_{r}+\gamma_{a})+\mu^{2}/\gamma_{m}}|^{2} \\ =|\frac{\gamma_{r}-\gamma_{a}}{\gamma_{r}+\gamma_{a}}|^{2}-|\frac{\gamma_{r}-\gamma'_{a}}{\gamma_{r}+\gamma'_{a}}|^{2} \end{matrix}$$

which is shown in Figure 3(d) of the main text.

S3. The absorption spectra and TCMT fitting results of meta-pixels with different geometric parameters.

**Figure S3 The absorption spectra and TCMT fitting results of meta-pixels with different geometric parameters.** The absorption spectra of meta-pixels with different geometric parameters: (a) height *h*, (b) length *L* and (c) period *p*. TCMT-fitted loss rates of meta-pixels with different geometric parameters: (d) height *h*, (e) length *L* and (f) period *p*.

S4. The effect of the resonator width *w* on losses.

**Figure S4 The effect of the resonator width *w* on losses.** (a) Absorption spectra of different *w*. (b) Extracted loss of different *w*.

S5. Resonance parameters extracted by TCMT fitting.

**Table S1 Extracted resonance parameters for different *h***

| *h* (μm) | Absorption | Resonant Frequency (THz) | *γ*_r_ (GHz) | *γ*_a_ (GHz) | *f* |
| --- | --- | --- | --- | --- | --- |
| 10 | 0.30773 | 1.166 | 26.53694 | 289.4336 | 0.09169 |
| 20 | 0.68951 | 1.1428 | 57.91778 | 203.68748 | 0.28435 |
| 30 | 0.90916 | 1.1116 | 78.25703 | 145.78315 | 0.5368 |
| 40 | 0.98991 | 1.0864 | 91.58459 | 112.03776 | 0.81744 |
| 50 | 0.99832 | 1.0668 | 97.89107 | 90.19034 | 1.08538 |
| 60 | 0.98374 | 1.052 | 96.82458 | 74.9233 | 1.29232 |

**Table S2 Extracted resonance parameters for different *L***

| *L* (μm) | Absorption | Resonant Frequency (THz) | *γ*_r_ (GHz) | *γ*_a_ (GHz) | *f* |
| --- | --- | --- | --- | --- | --- |
| 80 | 0.85968 | 1.1708 | 44.85587 | 98.59041 | 0.45497 |
| 84 | 0.88445 | 1.1492 | 58.69692 | 119.15455 | 0.49261 |
| 88 | 0.9012 | 1.1248 | 72.04544 | 138.10131 | 0.52169 |
| 92 | 0.91608 | 1.0984 | 83.87747 | 152.29295 | 0.55076 |
| 96 | 0.92781 | 1.07 | 92.9421 | 161.23567 | 0.57644 |
| 100 | 0.93557 | 1.0416 | 98.08083 | 164.81277 | 0.5951 |

**Table S3 Extracted resonance parameters for different *p***

| *p* (μm) | Absorption | Resonant Frequency (THz) | *γ*_r_ (GHz) | *γ*_a_ (GHz) | *f* |
| --- | --- | --- | --- | --- | --- |
| 300 | 0.91128 | 1.1135 | 81.47897 | 150.60737 | 0.541 |
| 304 | 0.90162 | 1.11 | 76.55602 | 146.52732 | 0.52247 |
| 308 | 0.8916 | 1.106 | 71.83097 | 142.34716 | 0.50462 |
| 312 | 0.88125 | 1.102 | 67.46767 | 138.41556 | 0.48743 |
| 316 | 0.87005 | 1.098 | 63.27146 | 134.6013 | 0.47007 |
| 320 | 0.85933 | 1.094 | 59.39736 | 130.69154 | 0.45449 |

**Table S4 Extracted resonance parameters for optimized frequency**

| *L* (μm) | *p* (μm) | *h* (μm) | *f* | Absorption | Resonant Frequency (THz) | *γ*_r_ (GHz) | *γ*_a_ (GHz) |
| --- | --- | --- | --- | --- | --- | --- | --- |
| 94 | 310 | 10 | 0.08705 | 0.29466 | 1.1112 | 26.80103 | 307.89381 |
| 95 | 310 | 20 | 0.27684 | 0.67923 | 1.11 | 58.13346 | 209.99082 |
| 90 | 301 | 30 | 0.53631 | 0.9089 | 1.1116 | 78.22769 | 145.86304 |
| 84 | 306 | 40 | 0.80041 | 0.98771 | 1.1112 | 83.69303 | 104.56206 |
| 84 | 300 | 50 | 1.06032 | 0.99914 | 1.1096 | 87.41913 | 82.44577 |
| 80 | 294 | 60 | 1.23954 | 0.98856 | 1.1092 | 83.86346 | 67.65712 |

**Table S5 Extracted resonance parameters for optimized absorption**

| d*L* (μm) | Absorption | Resonant Frequency (THz) | *γ*_r_ (GHz) | *γ*_a_ (GHz) | *f* |
| --- | --- | --- | --- | --- | --- |
| -10 | 0.90339 | 1.0305 | 84.16596 | 160.08436 | 0.52576 |
| -5 | 0.90521 | 1.0695 | 81.39463 | 153.8111 | 0.52919 |
| 0 | 0.90727 | 1.1095 | 76.40412 | 143.30972 | 0.53314 |
| 5 | 0.90342 | 1.15 | 68.27095 | 129.84013 | 0.52581 |
| 10 | 0.89845 | 1.1905 | 58.33495 | 112.90273 | 0.51668 |

S6. The effects of real and imaginary parts of the refractive index of the analyte on the metasensor resonance.

**Figure S5 The effects of real and imaginary part of the permittivity of the analyte on the metasensor resonance.**

S7. Ultra-broadband pixelated metasensor design.

**Figure S6 Resonance of the pixelated metasensor for ultra-broadband molecular fingerprints enhancement.**

**Table S6 Parameter settings of the ultra-broadband pixelated metasensor design.**

| Index | *p*_0_/μm | *L*_0_/μm | *S*/μm | d*L*/μm | *f/*THz |
| --- | --- | --- | --- | --- | --- |
| 1 | 350 | 190 | 0.1 | 0~40 | 0.50~0.60 |
| 2 | 350 | 190 | 0.1 | 0~28 | 0.6~0.69 |
| 3 | 350 | 160 | 0.3 | 0~24 | 0.7~0.81 |
| 4 | 342 | 134 | 0.6 | 0~24 | 0.82~0.96 |
| 5 | 324 | 108 | 1.2 | 0~16 | 0.97~1.10 |
| 6 | 305 | 90 | 1.8 | 0~18 | 1.10~1.27 |
| 7 | 278 | 72 | 2.5 | 0~20 | 1.28~1.53 |
| 8 | 234 | 52 | 3.0 | 0~16 | 1.54~1.85 |
| 9 | 190 | 36 | 3.4 | 0~10 | 1.86~2.17 |
| 10 | 157 | 26 | 4.2 | 0~6 | 2.19~2.52 |

S8. Optimization of the metasensor for glutamic acid vibrational enhancement.

Figure S7 Optimization of the metasensor for glutamic acid sensing. (a) Extracted loss parameters with different *h*. (b) Calculated (Theory) and simulated enhanced molecular signals as a function of loss rate *f*. (c) Simulated absorption spectra of the metasurfaces with/without a 2-μm-thick glutamic acid layer at different loss rate *f*.

S9. Absorption spectra of the metasensor and its 2D counterpart with different thickness of the glutamic acid layer coating.

**Figure S8 Absorption spectra of the metasensor with different thickness of the glutamic acid layer coating.**

Figure S9 Absorption spectra of the 2D in-plane metasensor with different thickness of the glutamic acid layer.

Figure S10 Extracted molecular signal enhancement as a function of molecule thickness.

S10. Strong coupling system analysis.

Based on the theory of coupled harmonic oscillators, the Hamiltonian of the hybrid system can be expressed as:^[4]^

$$H=\left(\begin{matrix} \omega_{\text{ph}} & g \\ g & \omega_{vib} \end{matrix}\right)$$

where $\omega_{ph}$ and $\omega_{vib}$ denote the complex frequencies of the metasurface resonance and the molecular vibration mode, respectively, and *g* represents the coupling strength. The eigenvalues of this Hamiltonian correspond to the frequencies of the upper (UPB) and lower (LPB) polariton branches:

$$\omega_{\pm} = \frac{\omega_{ph} +\omega_{\text{vib}} }{2} \pm\frac{1}{2}\sqrt{4g^{2}+\text{Δ}^{2} }$$

where $\text{Δ}=\omega_{\text{ph}}-\omega_{vib}$is the detuning. At zero detuning (Δ=0), the energy splitting between UPB and LPB reaches its minimum value, given by 2*g*, which defines the Rabi splitting strength:

$$\Omega=\sqrt{4g^{2}\text{-Δ}^{2}}$$

To confirm that the system operates in the strong coupling regime, the Rabi splitting must exceed the combined linewidths of the bare resonances, i.e.,

$$\Omega>\frac{\gamma_{ph}+\gamma_{vib}}{2}$$

where *γ*_ph_ and *γ*_vib_ are the damping rates of the photonic and vibrational modes. This criterion ensures that the hybridization dominates over dissipative losses, allowing well-defined polariton states to emerge. In this regime, the eigenstates are hybrid modes formed by a coherent superposition of the bare resonances. For example, the UPB state can be expressed as:

$$|UPB\rangle=a_{ph}|ph\rangle+a_{vib} |vib\rangle$$

where the square of *a*_ph_ and *a*_vib_ are the Hopfield coefficients, which represents the metasurface resonance mode and molecular vibration mode contributions and satisfies the normalization condition. They can be calculated by the following formula:

$$\begin{matrix} a_{ph}^{2}=\frac{1}{2} (1+\frac{\text{Δ}}{\sqrt{(\text{Δ})^{2}+4g^{2}}} ) \\ a_{vib}^{2} =\frac{1}{2} (1-\frac{\text{Δ}}{\sqrt{(\text{Δ})^{2}+4g^{2}}} ) \end{matrix}$$

For the LPB, the expressions are interchanged. Besides, it can also be noticed that the condition |*a*_ph_|^2^ + |*a*_vib_|^2^ = 1 is satisfied in all cases, as it meets the normalization condition. By evaluating these coefficients, one can quantitatively determine the photonic and vibrational fractions of the polariton states under different detuning and coupling conditions, thereby providing detailed insights into the hybridization mechanism in vibrational strong coupling systems.

S11. Comparison of different metasensors for molecular vibrational sensing.

**Table S7 Comparison of different metasensors for molecular vibrational sensing.**

| Ref.  year | Frequency band | Structure | Frequency  range | Optimization strategy | | Electric field enhancement  \|**E**/**E**_0_\|^2^ | Vibrational strong coupling realization | Sensing analytes and signal enhancement factor |
| --- | --- | --- | --- | --- | --- | --- | --- | --- |
|  |  |  |  | Frequency overlap | Coupling strength optimization |  |  |  |
| [1]  2018 | MIR | Dielectric tilted ellipse pairs | 1400–1800 cm^−1^ | √ | × | ∼1500 | × | Protein, 60 times |
| [2]  2019 |  | Crooked nanoantennas | - | × | √ | ∼10^3^ | × | PMMA, 25 times |
| [3]  2022 |  | Cross shaped resonance with MIM configuration | - | × | √ | >4000 | × | CO_2_, 4.2 times |
| [4]  2022 |  | Hook nanoantennas | 6–9 μm | √ | √ | 832891 | × | Silk, PMMA, ∼10 times |
| [5]  2023 |  | Tilted ellipse pairs with MIM configuration | 950–2000 cm^−1^ | √ | √ | >10^4^ | × | Protein, 3 times |
| [6]  2024 |  | Dielectric tilted ellipse pairs | 5.5–7.5 μm | √ | √ | NA | × | PMMA, NA |
| [7]  2024 |  | Dielectric tilted ellipse pairs | 1000–3200 cm^−1^ | √ | × | ∼900 | √ | PMMA, NA |
| [8]  2023 | THz | Symmetric crossed-slot resonator | 0.95–2.0 THz | √ | × | >10^5^ | × | chiral carnitine, 7.3 times |
| [9]  2024 |  | Inverted all-dielectric metagrating | 0.5–0.7 THz | √ | × | NA | × | α-lactose, 9.3 times |
| [10]  2024 |  | Complementary split-ring resonator | 0.6–1.2 THz | √ | × | NA | × | L-tyrosine, NA |
| [11]  2024 |  | Metagrating | 0.50–0.55 THz | √ | × | NA | × | α-lactose, 43 times;  Ta2O5, 98 times |
| [12]  2024 |  | Split ring resonator | 1.1–1.7 THz | √ | × | NA | √ | BST thin films |
| [13]  2024 |  | Planer double split-ring resonator | 1.5–2.3 THz | √ | × | ∼19044 | × | Glucose, 10.4 times |
| [14]  2025 |  | Planer split-ring resonator | 0.18–1.20 THz | √ | × | NA | × | L-tyrosine, 10 times |
| **This work** |  | **Cross shaped resonance with an out-of-plane configuration** | **0.5**–**2.5 THz** | **√** | **√** | **∼10^4^** | **√** | **Maltose, 12.6 times** |

**References**

[1] Tittl, A. Leitis, M. Liu, F. Yesilkoy, D.-Y. Choi, D. N. Neshev, Y. S. Kivshar, H. Altug, ***Science*** 2018, *360*, 1105.
[2] J. Wei, Y. Li, Y. Chang, D. Md. N. Hasan, B. Dong, Y. Ma, C.-W. Qiu, C. Lee, ***ACS Appl. Mater. Interfaces*** 2019, *11*, 47270.
[3] H. Zhou, D. Li, Z. Ren, X. Mu, C. Lee, ***InfoMat*** 2022, *4*, e12349.
[4] Z. Ren, Z. Zhang, J. Wei, B. Dong, C. Lee, ***Nat. Commun.*** 2022, *13*, 3859.
[5] J. Wang, T. Weber, A. Aigner, S. A. Maier, A. Tittl, ***Laser Photonics Rev.*** 2023, *17*, 2300294.
[6] A. Aigner, T. Weber, A. Wester, S. A. Maier, A. Tittl, ***Nat. Nanotechnol.*** 2024, *19*, 1804.
[7] F. U. Richter, I. Sinev, S. Zhou, A. Leitis, S. Oh, M. L. Tseng, Y. Kivshar, H. Altug, ***Adv. Mater.*** 2024, *36*, 2314279.
[8] J. Lyu, S. Shen, L. Chen, Y. Zhu, S. Zhuang, ***PhotoniX*** 2023, *4*, 28.
[9] X. Liu, Y. Xie, Y. Yan, Q. Niu, L.-G. Zhu, Z. Dong, Q. H. Liu, J. Zhu, ***ACS Photonics*** 2024, *11*, 4838.
[10] Li, X. Xiang, P. Wang, Y. Teng, H. Chen, W. Li, S. Yang, B. Chen, C. Zhang, J. Wu, K. Fan, B. Jin, L. Jiang, J. Chen, P. Wu, ***Opt. Express*** 2024, *32*, 27473.
[11] Y. Xie, X. Liu, J. Zhou, H. Zhang, J.-Y. Lin, W. Chen, L.-G. Zhu, K. Meng, Q. H. Liu, J. Zhu, ***IEEE Trans. Microwave Theory Techn.*** 2024, *72*, 2368.
[12] S. M. Mekonen, D. Jain, S. Oh, N. P. Armitage, ***ACS Photonics*** 2024, *11*, 2242.
[13] W. Han, P. Wang, C. Zhang, Y. Xu, Z. Wu, Q. Wang, ***ACS Appl. Mater. Interfaces*** 2024, *16*, 70795.
[14] H. Sun, Y. Cao, Y. Chen, L. Li, L. Ma, S. Jin, G. Gu, X. Song, W. Wu, Z. Feng, ***ACS Photonics*** 2025, *12*, 5302.
S12. Schematic diagram of PμSL-based 3D printing technique.

**Figure S11 Schematic diagram of PμSL-based 3D printing technique.** PμSL is a 3D printing technology based on area projection triggered photopolymerization, and capable of fabricating complex 3D architectures covering multiple scales and with multiple materials.

S13. Optical path of the THz-TDS.

**Figure S12 Schematic diagram of the THz-TDS system.** The optical path includes the femtosecond laser, the time delay line, the reflection measurement module, the scanning system, and the control module.

**References**

1. C. Yang, Z. Shen, Y. Cui, et al., “Terahertz Molecular Vibrational Sensing Using 3D Printed Anapole Meta-Biosensor,” *Biosensors & Bioelectronics* *278* (2025): 117351, https://doi.org/10.1016/j.bios.2025.117351.

2. S. Fan, W. Suh, and J. D. Joannopoulos, “Temporal Coupled-Mode Theory for the Fano Resonance in Optical Resonators,” *Journal of the Optical Society of America A : OPTICS IMAGE SCIENCE AND VISION* *20*, no. 3 (2003): 569, https://doi.org/10.1364/JOSAA.20.000569.

3. W. Suh, Z. Wang, and S. Fan, “Temporal Coupled-Mode Theory and the Presence of Non-Orthogonal Modes in Lossless Multimode Cavities,” *IEEE Journal of Quantum Electronics* *40*, no. 10 (2004): 1511–1518, https://doi.org/10.1109/JQE.2004.834773.

4. P. G. Zotev, P. Bouteyre, Y. Wang, et al., “Nanophotonics with Multilayer van Der Waals Materials,” *Nature Photonics* *19*, no. 8 (2025): 788–802, https://doi.org/10.1038/s41566-025-01717-x.
